# When nanoparticles meet biofilms—interactions guiding the environmental fate and accumulation of nanoparticles

**DOI:** 10.3389/fmicb.2015.00591

**Published:** 2015-06-16

**Authors:** Kaoru Ikuma, Alan W. Decho, Boris L. T. Lau

**Affiliations:** ^1^Department of Civil and Environmental Engineering, University of Massachusetts Amherst, Amherst, MA, USA; ^2^Department of Environmental Health Sciences, University of South Carolina, Columbia, SC, USA

**Keywords:** nanoparticle–biofilm interactions, extracellular polymeric substances, protein corona, pore space, surface forces, biofilm matrix

## Abstract

Bacteria are essential components of all natural and many engineered systems. The most active fractions of bacteria are now recognized to occur as *biofilms*, where cells are attached and surrounded by a secreted matrix of “sticky” extracellular polymeric substances. Recent investigations have established that significant accumulation of nanoparticles (NPs) occurs in aquatic biofilms. These studies point to the emerging roles of biofilms for influencing partitioning and possibly transformations of NPs in both natural and engineered systems. While attached biofilms are efficient “sponges” for NPs, efforts to elucidate the fundamental mechanisms guiding interactions between NPs and biofilms have just begun. In this mini review, special attention is focused on NP–biofilm interactions within the aquatic environment. We highlight key physical, chemical, and biological processes that affect interactions and accumulation of NPs by bacterial biofilms. We posit that these biofilm processes present the likely possibility for unique biological and chemical transformations of NPs. Ultimately, the environmental fate of NPs is influenced by biofilms, and therefore requires a more in-depth understanding of their fundamental properties.

## Introduction: Important Properties of the Biofilm Microenvironment in the Bigger Picture

Microbial biofilms are an omnipresent component in many environments supporting life. Hence, the multifaceted roles of biofilms in the sequestration, accumulation, transformation, and trophic transfer of environmental contaminants have been a subject of much study and controversy. A biofilm is, in its simplest form, a collection of surface-attached microbial cells that are surrounded by a matrix of extracellular polymers. The inherent properties and physical structure of biofilms resemble that of a sorptive sponge capable of capturing various chemical and biological components in their vicinity. Natural and engineered systems that are significantly impacted by biofilms include soil mineral surfaces, microbial mats, wastewater treatment, and biofouling of ships and pipes. These will not be addressed further here. Rather, this review focuses on the mechanistic interactions of bacterial biofilm with natural and synthetic nanoparticles (NPs); an emerging concern in both the environment and health. Such NP–biofilm interactions within the aquatic environment are highlighted.

## Nanoparticle–Biofilm Interactions

It is now recognized that environmental biofilms are efficient binding matrices for NPs (Battin et al., 2009; Ferry et al., 2009; Nevius et al., 2012; Kroll et al., 2014), and this can be attributed largely to the extracellular polymeric substances (EPS) that hold biofilms together (Flemming and Wingender, 2010; Nevius et al., 2012). Recent studies have shown that significant accumulations of NPs occurred in biofilms of riverine- and marine-mesocosms (Battin et al., 2009; Ferry et al., 2009). When gold nanorods (65 nm × 15 nm) were added to a marine/estuarine mesocosm containing sediments, seagrass, bivalves, shrimp, and plankton, the nanorods were most strongly bioconcentrated by microbial biofilms with their bioconcentration accounting for greater than 60% of the added nanorods (Ferry et al., 2009). Similar bioconcentration was found in riverine mesocosms using 20 nm TiO_2_ NPs (Battin et al., 2009). These initial studies point to an important role of biofilms for influencing environmental partitioning of NPs within natural systems. In retrospect, this is not surprising since biofilms are efficient chelators for physical-trapping and binding of dissolved and colloidal forms of metals and organic matter in a wide range of systems such as wastewater treatment (Wuertz et al., 2000; Hu et al., 2005; Hawari and Mulligan, 2006), drinking-water filtration (Lehtola et al., 2004; Berry et al., 2006), and marine and freshwater systems (Schlekat et al., 1998; Decho, 2000; Battin et al., 2009). As the research focus on NP–biofilm interactions is still in its early stages, this mini review is designed to provide a brief overview of published studies and some insights into future directions to improve our understanding of the mechanisms and the bigger-picture implications of these interactions.

**FIGURE 1:**
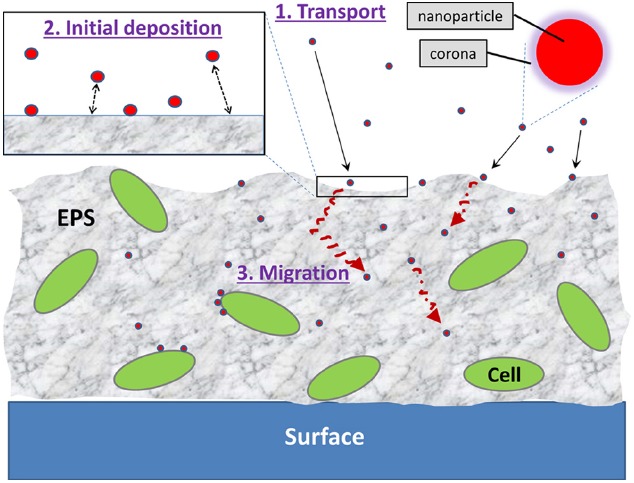
**The three steps involved in NP–biofilm interactions: (1) transport of NPs to the vicinity of the biofilm, (2) initial deposition of NPs onto the biofilm surface, and (3) migration of NPs into deeper areas of the biofilm.** NPs may also interact directly with cell surfaces within the biofilm matrix.

The interactions between NPs and the biofilm can be viewed as a three-step process: (1) *transport of NPs to the vicinity of the biofilm*; (2) *attachment to the biofilm surface*; and (3) *migration within the biofilm* (Figure 1). At each of these steps, the interactions are a complex interplay of a myriad of factors including, but not limited to, NP characteristics, the physicochemical and biological makeup of the biofilm matrix, and environmental parameters such as water chemistry, flow, and temperature. The effects of various environmental parameters on the fate of NPs have been extensively studied and reviewed in detail (e.g., Petosa et al., 2010; Levard et al., 2012). Similarly, many biofilm researchers have studied how environmental parameters influence biofilms (as reviewed by, e.g., Sutherland, 2001; Karatan and Watnick, 2009). For example, the effects of ionic strength of the aqueous environment on both NPs (e.g., NPs aggregate as ionic strength increases) and biofilms (e.g., pore sizes change with different ionic strengths) are well documented. While such parameters are likely to have direct or indirect impacts, studies that thoroughly examine their influence on NP–biofilm interactions are currently lacking. Rather, the following sections aim to highlight the currently known NP and biofilm factors that have a critical impact on their interactions.

### Impact of NP Characteristics on NP–Biofilm Interactions

Engineered and naturally forming NPs can vary widely in their physicochemical characteristics such as shape, size, and charge (Hochella et al., 2008; Petosa et al., 2010). As discussed in the following section, these NP characteristics have been reported to impact their interactions with biofilm-coated surfaces at all three steps of the interactions. NP transport through the water column has been studied extensively (Lecoanet and Wiesner, 2004; Jaisi et al., 2008; Phenrat et al., 2009) and is primarily a function of various NP characteristics and water chemistry conditions; these bulk-phase transport phenomena will not be discussed in length here. Instead, this section will focus on the NP characteristics that influence the small-scale interactions in the proximity of biofilm surfaces including transport and attachment.

Surface modification of NPs, through ligand capping during synthesis or post-synthesis passive sorption of organic molecules, plays a critical role in NP–biofilm interactions. In fact, pure and single-component NPs are rare or non-existent in the environment. Engineered NPs are typically functionalized by specific organic ligands for a variety of target applications. When these capped NPs are being used or released in various environments, they are often subjected to further modifications in an uncontrollable manner by passive sorption of different organic molecules (e.g., proteins and polysaccharides). Dawson and colleagues introduced the concept of the “protein corona” as an important entity for NPs interacting with the external environment (Cedervall et al., 2007; Lynch et al., 2009; Walczyk et al., 2010). The corona is a temporally evolving collection of organic molecules that associate with NPs. Though these initial studies focused on proteins, recent investigations have expanded the concept of the “corona” to include other biomolecules as well as proteins (Monopoli et al., 2012). When a NP with an organic corona approaches a surface, proteins and other biomolecules that reside long enough on the NP surface will mediate subsequent interactions. These biomolecule–NP interactions are highlighted particularly in nanomedicine as a biofunctionalization mechanism. In environmental systems, a corona-like coating is also likely to form around NPs; in this case, the organic molecules in the corona are expected to primarily consist of components of natural organic matter (NOM). The organic corona as well as other organic coatings of NPs are likely to have significant impacts on NP–biofilm interactions. For example, cadmium selenide quantum dots (QDs) conjugated with polyethylene glycol were found to penetrate more easily into *Pseudomonas aeruginosa* PAO1 biofilms than QDs having surface carboxyl (–COOH) groups (Morrow et al., 2010). In another study, incubation of *Pseudomonas fluorescens* biofilms with silver NPs pre-exposed to NOM was shown to result in greater cell viability compared to silver NPs without NOM exposure (Wirth et al., 2012), suggesting that the NOM-based corona associated with the NPs had a mitigating effect on silver toxicity.

The charge and size of NPs also affect NP–biofilm interactions. In the case of fluorescent polystyrene NPs, surface sulfate (SO^4–^) groups on NPs resulted in greater sorption onto *Alteromonas macleodii* biofilms compared to NPs functionalized with amine (–NH) or carboxyl groups (Nevius et al., 2012). Both the size and charge of silver NPs were reported to be important in modulating their transport within *P. fluorescens* biofilms with their self-diffusion coefficients decreasing with increasing size and negative charge (Peulen and Wilkinson, 2011). This effect of NP size was also dependent on the density of the biofilm with NP self-diffusion becoming severely limited when the size was larger than 50 nm only in dense biofilms. Electrostatic (not steric) forces were shown to control the diffusion of positively and negatively charged latex beads (∼28 nm) in biofilms having relatively low (*Lactococcus lactis*) and high (*Stenotrophomonas maltophilia*) EPS contents (Guiot et al., 2002). Furthermore, particle size, charge, and particle surface chemistry may collectively affect the fate and transport of NPs in biofilm-coated porous media (Tripathi et al., 2012). While it is clear that NP characteristics influence NP–biofilm interactions, these interactions involve a highly complex interplay of such characteristics as well as biofilm features.

### Impact of Biofilm Matrix Chemistry on NP–Biofilm Interactions

The EPS matrix is the primary emergent property of the biofilm (Flemming and Wingender, 2010). Once NPs reach the water–biofilm interface, the physicochemical matrix of EPS has direct implications on both the initial attachment of NPs onto the biofilm surface and their subsequent movement into the biofilm matrix. The EPS matrix is physicochemically complex and extremely heterogeneous over small spatial scales (e.g., micrometers; Lawrence et al., 2007). It can be thought of as a 3D filter, which surrounds biofilm cells and forms a dynamic trapping network for organic molecules and ions, and NPs, and consists of an interlinked network of polymer molecules, many of which are charged.

The density of EPS depends upon its local concentration, but also the charges and number of linkages between adjacent polymer chains. Although the EPS matrix is usually highly hydrated (often 99 % wt/wt), most of the water is not bound to EPS but rather is localized in pore spaces between adjacent polymer chains (Schmitt et al., 1995). These physicochemical features of EPS significantly impact NP–biofilm interactions. Of particular interest is how different molecules within the EPS matrix influences the initial deposition and continued accumulation of NPs. EPS are a complex array of polysaccharides, proteins, lipids, and even nucleic acids (Whitchurch et al., 2002; Flemming and Wingender, 2010). Functional group moieties on individual EPS molecules have varying potentials to bind ions, charged molecules, and NPs (Braissant et al., 2009). The nature of linkages between an EPS functional group(s) and the sorbed moiety (e.g., NPs), therefore, can result in binding with different affinities during initial deposition and subsequent accumulation.

While the initial attachment of NPs onto the outermost surface of biofilms may be influenced by a variety of physicochemical interactions, the specifics of these interfacial NP–biofilm interactions are largely unknown. A recent study by our laboratory showed that surfaces coated with polysaccharides, a major component of EPS, significantly affected the deposition of iron oxide NPs (hematite, α-Fe_2_O_3_) NPs (Ikuma et al., 2014). Different physicochemical features of surface-adsorbed polysaccharides, particularly surface charge heterogeneity, resulted in varying degrees of NP deposition due to changes in electrostatic interactions (see Liang et al., 2007 for a review of intermolecular forces). These observations strongly indicate that, unsurprisingly, not all polysaccharides (or any other group of EPS components) are equal for NPs, and thus, simple chemical characterization of the biofilm matrix into groups such as polysaccharides and proteins may not provide the necessary information for assessing the likelihood of the occurrence of NP–biofilm interactions. Electrostatic forces were also implicated as an important mechanism for deposition of TiO_2_ NPs onto synthetic biofilms (Sahle-Demessie and Tadesse, 2011) and for fullerene (C_60_) NPs onto surfaces coated with EPS extracted from *Escherichia coli* (Tong et al., 2010). On the other hand, polymer-mediated steric interactions were suggested as a dominant force for the attachment of poly(acrylic acid)-stabilized zerovalent iron NPs onto biofilm-coated porous media (Lerner et al., 2012). While this observation was based on NPs with polymer coatings, steric interactions are likely to be important in NP–biofilm interactions in the aquatic environment due to the polymeric natures of biofilms, and NPs coated with an organic corona. In addition, other potential interaction forces involved in NP deposition onto biofilm surfaces can be inferred from recent studies showing NP attachment to surfaces coated with organic compounds. For example, NOM has been extensively documented to adsorb to various NPs through combinations of many forces such as electrostatic, steric, and hydrophobic interactions (e.g., Hyung et al., 2007; Pelley and Tufenkji, 2008; Stankus et al., 2011).

Once a NP binds to EPS, it can subsequently migrate deeper into the EPS matrix. NP penetration into and movement within the biofilm is considered to be driven primarily by diffusion (Peulen and Wilkinson, 2011). In this case, diffusion of NPs into the biofilm may depend on the pore sizes of the biofilm (Sahle-Demessie and Tadesse, 2011), the charge of both the NPs and the biofilm matrix (Peulen and Wilkinson, 2011), hydrophobicity of the surrounding environment (Habimana et al., 2011), and the chemical gradient within the matrix. The EPS matrix pore-spaces (containing water) between adjacent molecules can vary in size. Ions and organic molecules diffuse and penetrate into a biofilm by moving (i.e., diffusing) through these pore-spaces. This presents the likely possibility that the EPS pore-spacing will be especially important in this process. This nanoscale variability, however, is poorly characterized and understood.

While accumulation of NPs within biofilms results from initial attachment and migration of NPs, these two processes need not always occur sequentially. It is likely that more NPs are depositing onto the outermost surface simultaneously as other NPs are penetrating into the biofilm matrix and vice versa. Another possible mechanism that contributes to NP accumulation within the matrix is the active outgrowth of the biofilm, forming new layers above the surface-deposited NPs. In such cases, penetration of NPs into the biofilm matrix is not necessary to occur for accumulation to take place. However, differentiation between these different processes would be a difficult task in practice. All three are likely to occur in the complex and dynamic environments where biofilms naturally occur. Most recent studies on NP–biofilm interactions have examined either the combined effects of initial surface NP deposition and penetration into the biofilm (Morrow et al., 2010; Habimana et al., 2011; Peulen and Wilkinson, 2011) or all three steps of NP–biofilm interactions outlined above in Section “Nanoparticle–Biofilm Interactions” (transport, attachment, migration; Fabrega et al., 2009; Choi et al., 2010). Overall, the migration of NPs into the 3D matrix of the biofilm is the least understood step of NP–biofilm interactions.

## Fate of Nanoparticles Within Biofilms

Accumulation of NPs within biofilms has been previously documented (Ferry et al., 2009; Fabrega et al., 2011). Is the biofilm a “sink” for concentrating NPs from the overlying water? This is not likely to be the case. A final point here is that since biofilms and their associated EPS are readily consumed by grazing animals (see Decho, 1990, 2000, for reviews), the biofilm presents a potentially efficient vehicle for the trophic-transfer of NPs to food webs. However, the exact fate of the NPs within biofilms is not clear.

### Behavior of Nanoparticles Upon Accumulation

One question we could ask is whether the NPs stay nano-sized and as particles. Given the right conditions, NPs will easily aggregate to form micro-size agglomerates. As shown by Choi et al. (2010), nanosilver that is introduced into *E. coli* biofilms were shown to aggregate to a larger degree than in planktonic cultures, possibly due to differences in ionic strength used in the experiments. On the other hand, organic ligands, including some that are found in EPS, typically stabilize NPs against aggregation, suggesting that NPs that enter the biofilm matrix as monomers may indeed stay as such within the biofilm. Another potential outcome of NP accumulation is NP dissolution as the NPs may be surrounded by enzymes and other organic matter that speed up those processes. This may especially be an important outcome for metallic NPs. Carbon-based NPs may undergo different changes in biofilms compared to metallic NPs. For example, microbial transformation of carbon-based NPs has been documented (Chae et al., 2014), which may also occur in the biofilm if the NPs are in contact with cells.

The EPS matrix is the primary emergent- and adaptive-property of the microbial cells forming a biofilm (Flemming and Wingender, 2010). Therefore, the matrix may potentially change in response to the presence of NPs. NP effects on biofilm microorganisms are likely to be dependent on the type of NP that is accumulated. For example, in the case of nanosilver or other NPs with antibacterial effects, cells could be severely stressed or directly killed as the concentration of NPs embedded in the biofilm increase. However, these antibacterial effects of nanosilver appeared to be considerably lower on cells in biofilms compared to planktonic cells (Choi et al., 2010). Other antibacterial NPs are designed to overcome such barriers; for example, Hetrick et al. (2009) developed nitric oxide-releasing silica NPs that do not rely on direct NP–cell contact, and hence, appeared to be highly effective at controlling pathogenic biofilm growth. The targeted use of NPs for biofilm control has been studied extensively by medical and dental as well as biofouling researchers and has been reviewed in detail (Allaker, 2010; Sousa et al., 2011; Natalio et al., 2012). Furthermore, nanosilver has been shown to affect the microbial community in wastewater biofilms (Sheng and Liu, 2011). These changes in the microbial community structure were mainly attributed to the antibacterial effects of nanosilver and the differences in tolerance levels across bacterial species. On the other hand, even NPs that have no antibacterial effects may induce shifts in the biofilm microbial community possibly due to changes in nutrient availability or chemical gradient within the biofilm matrix (Fabrega et al., 2011).

### Formation of NPs Within Biofilms

The potential of biofilms to act as a factory for NP production has been increasingly recognized in both natural and engineered systems. Natural biofilms play a critical role in the biogeochemical cycling of elements which can lead to NP formation. For example, microbes precipitate metals in the form of NPs as a detoxification mechanism. Reith et al. (2006, 2010) have shown that gold dissolution and re-precipitation of nanoparticulate gold is directly coupled with biofilms on gold grain surfaces. Biofilms dominated by sulfate reducing bacteria were found to be responsible for the formation of zinc sulfide NPs (Labrenz et al., 2000; Labrenz and Banfield, 2004). As the number of such biofilm-mediated NP formation studies increases, the use of biofilms in the synthesis of nanomaterials is becoming popular for its relatively clean, non-toxic, and environmentally benign procedures (Mandal et al., 2006). For example, an electrochemically active biofilm was being utilized as a catalyst for extracellular production of monodispersed crystalline silver NPs (Kalathil et al., 2011).

## Conclusions and Future Directions—Environmental Implications

There has been significant progress in understanding the micro- and nano-scale complexity within biofilms. Despite their complexity, it is now possible to experimentally examine the nature of NP interactions and penetration into biofilms. The ability to understand these fundamental and decisive processes can be approached using carefully controlled laboratory manipulations of EPS and NPs. Recent development of surface-sensitive techniques, high-resolution microscopies, and synchrotron-based spectroscopies provide powerful tools to promote an integrated approach to understanding NP–biofilm interactions. Multi-scale computational modeling efforts will be useful in complementing empirical data and enhancing the predictability of NP behavior within the biofilm matrix. Ultimately, improving our mechanistic understanding of NP–biofilm interactions will enable better risk assessment of nanotechnology as well as sustainable design of NPs.

### Conflict of Interest Statement

The authors declare that the research was conducted in the absence of any commercial or financial relationships that could be construed as a potential conflict of interest.
